# Analysis of removal of cadmium by action of immobilized *Chlorella *sp. micro-algae in alginate beads

**DOI:** 10.12688/f1000research.13527.1

**Published:** 2018-01-15

**Authors:** Christian Valdez, Yomaira Perengüez, Bence Mátyás, María Fernanda Guevara

**Affiliations:** 1 Carrera de Ingeniería en Biotecnología de los Recursos Naturales, Universidad Politécnica Salesiana, Quito, Ecuador; 2 Grupo de Investigación Mentoria y Gestión del Cambio, Universidad Politécnica Salesiana, Cuenca, Ecuador; 3 NUNKUY-WAKAN: Environmental, Secondary Metabolites and Animal Biotechnology Research Group, Universidad Politécnica Salesiana, Quito, Ecuador

**Keywords:** Chlorella sp., cadmium removal, immobilization, alginate

## Abstract

Cadmium (Cd) is a metal that can negatively interfere with the metabolic systems of living beings. The objective of this work was to evaluate the capacity for cadmium removal in aqueous solutions by immobilized *Chlorella *sp. in calcium alginate beads. Beads without *Chlorella* sp. were used as a control. All the treatments were established in triplicate for 80 min, at four concentrations of cadmium (0, 20, 100 and 200 ppm), taking samples of aqueous solution every 10 min, to be read using atomic absorption equipment. The study determined that the treatment of alginate beads with immobilized *Chlorella* sp. removed 59.67% of cadmium at an initial concentration of 20 ppm, this being the best removal result.

## Introduction

Pollution from the use of metals as a consequence of waste generated by industries is a constant concern, since they may end up being transferred to the environment ^1, 2^. The need to develop technologies for the remediation of water contaminated with cadmium should be considered as a priority in Ecuador. The objective of this study was to evaluate the capacity for cadmium removal in aqueous solutions by immobilized *Chlorella* sp. in calcium alginate beads. This study used the micro-algae *Chlorella* sp., for the bio-removal of cadmium, since the use of cells of *Chlorella* sp. immobilized in beads of alginate has been successfully exploited ^3^ and its potential use has been established in the bioremediation of contaminants such as metals ^4^, nutrients ^5^ and other industrial pollutants ^6^.

## Methods

### Alginate beads

The assay was carried out with aqueous solutions of cadmium. This solution was placed together with the alginate beads in Florence flasks. Every 10 minutes a sample of the aqueous solution was taken (10 mL approximately) with a glass pipette, and each sample was placed in a test tube. The concentration of metal was measured by aspiration atomic absorption spectrophotometer.

To make the control beads, the procedure described in a previous work was followed ^7^. Briefly, 2 grams of sodium alginate (Loba Chemie, high density) were dissolved in 200 mL of ultra-pure water to obtain a homogeneous mixture, adjusting the pH between 7.8–8.0. pH was adjusted by addition of 0.1 molar sodium hydroxide or 0.1 molar hydrochloric acid, in sodium alginate paste (homogeneous mixture). One drop at a time was slowly poured into a 1% w/v calcium chloride solution with constant stirring.

For the immobilization of *Chlorella* sp. in alginate beads, liquid cultures were used. The volume of culture to be used was established to reach a concentration of 25 X 10 ^6^ cells mL per each mL of alginate. For the preparation of the beads, 2 g of sodium alginate were dissolved in microalgae culture, as above.

### Removal of cadmium with alginate beads

Experimental liquids were placed in 12 Florence Flasks of 500 mL capacity, inside which were placed 50 g of alginate beads without immobilized *Chlorella sp.* and 200 mL of cadmium solution at different concentrations (0, 20, 100 and 200 ppm). Each concentration was performed in triplicate, essay was evaluated during 80 minutes, aqueous solution samples were taken at 10 min intervals. Each Florence flask was placed on a shelf in the laboratory, the location of each bead on this surface was performed randomly. The flasks were subjected to constant aeration with air pressure of 0.012 MPa for 80 min. Every 10 minutes, an aqueous solution sample was taken to measure the concentration of metal present. These were the control treatments.

For the experimental samples, 50 g of alginate beads with immobilized *Chlorella* sp. were placed in each flask with the same concentrations of cadmium solution as the controls. These experiments were also performed in triplicate. These experimental samples were used to establish whether the addition of *Chlorella* sp. in the beads, increases or decreases the alginate removal activity.

The determination of metal concentration in the solutions was measured with a VARIAN direct aspiration atomic absorption spectrophotometer (VARIAN. Model: SpectrAA 55, Wavelength: 326.1 nm), using air-acetylene flame with cadmium lamp, and results are presented as % of removal of cadmium.

### Viability of micro-algae exposed to cadmium

For viability tests, color change of the cultures exposed to cadmium was evaluated. For this purpose 100 mL of liquid *Chlorella* cultures were used (25 × 10 ^6^ cells/mL) and they were subjected to different concentrations of Cd (0, 20, 100 and 200 ppm), in triplicate. The color change was monitored at the first hour of treatment, at 24 hours and at 7 days.

## Results and discussion

### Viability of micro-algae exposed to cadmium

**Figure 1.**
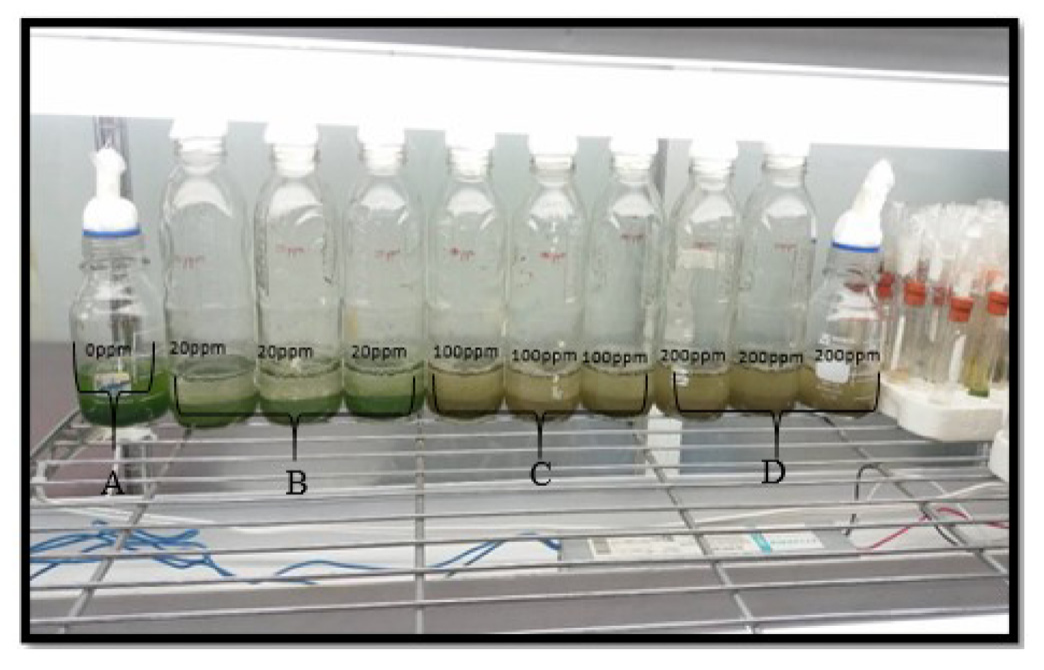
Viability tests of *Chlorella sp*. subjected to different concentrations of Cd, at 60 minutes, ( **A**) 0 ppm Cd, ( **B**) 20 ppm Cd, ( **C**) 100 ppm Cd, ( **D**) 200 ppm Cd. Green color means that algae remain viable, brown color means that *Chlorella* loses viability.

Color changes were observed in the solutions ( Figure 1, Dataset 1 and Dataset 2) of free micro-algae cells exposed to different concentrations of cadmium, which is a product of the toxicity of the metal, while the solutions without the presence of metal maintained a green color, characteristic of *Chlorella sp.* ^7^

It was determined that *Chlorella* sp. exposed to a concentration of 20 ppm cadmium at 60 minutes of contact does not react, and shows no noticeable changes in coloration. On the other hand, the micro-algae did not show resistance to concentrations 100 and 200 ppm of Cd, revealing that the cells did not remain viable due to the color variation and high toxicity of the metal used, going from green to a grayish brown in the first 60 minutes of the start of the trial. These results show to decrease algal growth and inhibit photosynthesis ^8^ 100 ppm or higher.

Percentage of Cd in aqueous solution at 80 minutes Click here for additional data file. Copyright: © 2018 Valdez C et al. 2018 Data associated with the article are available under the terms of the Creative Commons Zero "No rights reserved" data waiver (CC0 1.0 Public domain dedication).

Raw data for the percentage removal of cadmium with and without *Chlorella* alginate beads at all concentrations and triplicate experiments Click here for additional data file. Copyright: © 2018 Valdez C et al. 2018 Data associated with the article are available under the terms of the Creative Commons Zero "No rights reserved" data waiver (CC0 1.0 Public domain dedication).

### Removal of cadmium with alginate beads

**Figure 2.**
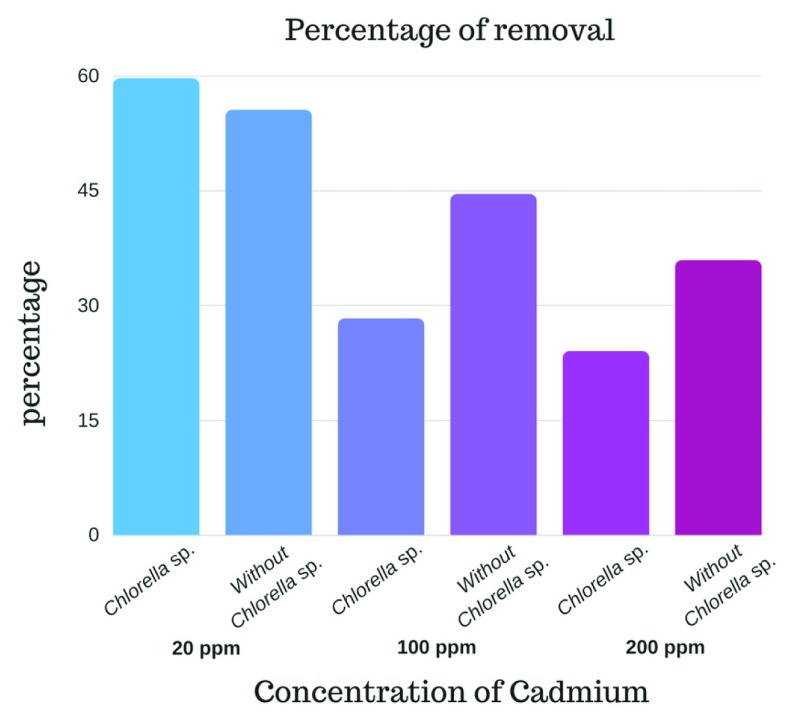
Comparison of the percentage of cadmium with different treatments carried out: alginate beads without *Chlorella sp*. (Without *Chlorella*), and alginate beads with *Chlorella sp*. ( *Chlorella*).

**Figure 3.**
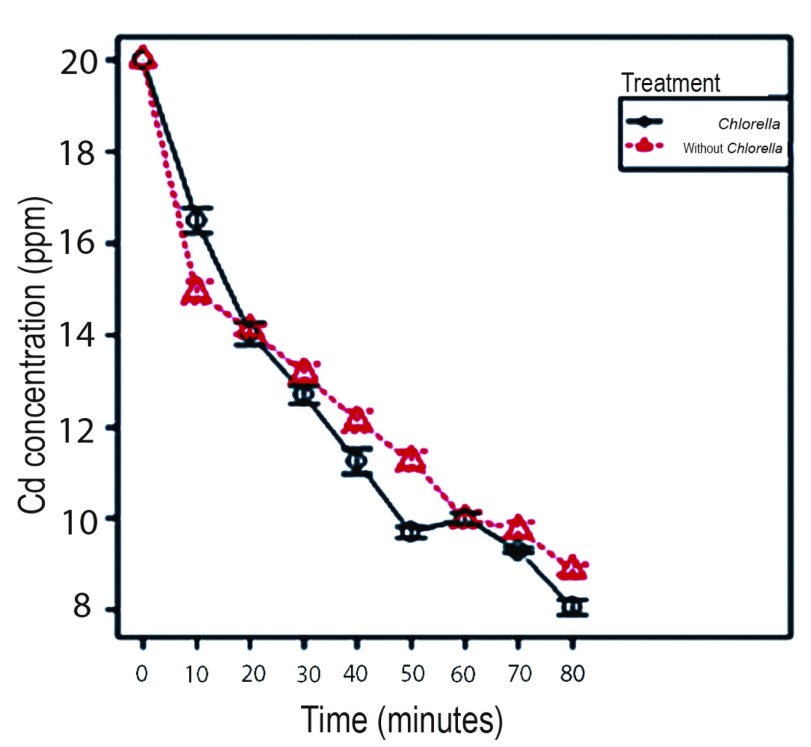
Variation of the concentration of Cd in the aqueous solution with respect to the time during the process of removal of 20 ppm of metal by the action of alginate beads with *Chlorella* sp. and without *Chlorella* sp.

It is verified that the best treatment for Cd removal is the one corresponding to alginate beads with immobilized *Chlorella* sp. at the concentration of 20 ppm of Cd at 80 min. It was determined that at low metal concentrations the micro-algae enhances its removal capacity achieving a removal percentage of 59.67% with *Chlorella* sp., being significantly higher (p value <0.001) than the removal presented without Chlorella sp. (55.56%), explained by the viability process that showed that *Chlorella* sp. can withstand the toxicity of the metal for periods of at least 60 min at concentrations of 20 ppm ( Figure 2 and Figure 3). As demonstrated by 9, micro-algae work better at lower concentrations of metal.

*Chlorella* sp. immobilized in alginate beads could be used for bioremediation processes of cadmium at low concentrations of the metal, since the presence of viable biomass of the micro-algae potentiates the removal capacity of the alginate. Non-viable *Chlorella sp.*, because of the high concentration of cadmium (100 and 200 ppm), decreases its removal capacity, yielding better results in beads treated without *Chlorella sp.* Thus, the alginate matrix without micro-algae could be used effectively in Cd removal processes at high metal concentrations.

## Data availability

The data referenced by this article are under copyright with the following copyright statement: Copyright: © 2018 Valdez C et al.

Data associated with the article are available under the terms of the Creative Commons Zero "No rights reserved" data waiver (CC0 1.0 Public domain dedication). 

Dataset 1. Percentage of Cd in aqueous solution at 80 minutes. DOI, 10.5256/f1000research.13527.d190266 ^10^

Dataset 2. Raw data for the percentage removal of cadmium with and without *Chlorella* alginate beads at all concentrations and triplicate experiments DOI, 10.5256/f1000research.13527.d190281 ^11^
