# Supplementary material for: Analysis of removal of cadmium by action of immobilized Chlorella sp. micro-algae in alginate beads
Source: F1000Res. 2018 Jan 15;7:54. [Version 1] doi: 10.12688/f1000research.13527.1 (PMC5785717; doi:10.12688/f1000research.13527.1)
Supplement: Peer review history

# Peer review: Referee response for version 1

**DOI:** 10.5256/f1000research.14689.r29834

**Published:** 2018-01-24

**Authors:** Bernadett Gálya

**Affiliations:** 1 Institute of Water and Environmental Management, University of Debrecen, Debrecen, Hungary

In my opinion, this article is well editable and easy to follow. The scope of each chapter is appropriate. In the Introduction, they briefly presented the problem behind the study. The study design is appropriate and the work is technically sound. They cited 11 references which is strongly connected to this topic. These are from 1998 to 2018, all in all, current literature was used. The method was described in detail, thus it is easy to replicate. They created three main parts in the methods. In my opinion is fine, but I suggest that the third subchapter should be the test used during the experiment. Otherwise, the materials, repetition and measurement which they are used are appropriate. The charts that are used are clear and easy to follow these in the text. In this article, the statistical analyses is applicable. May I suggest, that in the future they should use some other statistical test to compare different treatments (such as Tukey or Duncan-test). All source data are underlying the results available to ensure full reproducibility. The conclusions are drawn adequately supported by the results. Summary, this article is clear, easy to follow, used appropriate methods and scientifically based. Thus, it will be very useful to solve the examined problem.

I have read this submission. I believe that I have an appropriate level of expertise to confirm that it is of an acceptable scientific standard.

# Peer review: Referee response for version 1

**DOI:** 10.5256/f1000research.14689.r29838

**Published:** 2018-01-23

**Authors:** Juan Enrique Tacoronte Morales, Olinka Tiomno

**Affiliations:** 1 Centro de Biología, Proyecto Prometeo-SENESCyT, Universidad Central del Ecuador, Quito, Ecuador; 2 Center for Chemical Research and Engineering (CIIQ), Havana, Cuba

The problems of pollution with heavy metals is a serious environmental issue. The use of microalgae supported on different substrates is an eco-biotechnological variant of great importance in sustainable conditions. The work is clear, concise and develops a simple and applicable strategy in field and laboratory conditions. The utilization of alginate beds (Ca 2+) is a great technological solution

We have read this submission. We believe that we have an appropriate level of expertise to confirm that it is of an acceptable scientific standard.
